# Integrative Analysis of Genetic Risk Factors for Acute Myeloid Leukemia Using Mendelian Randomization and Single‐Cell RNA Sequencing Validation

**DOI:** 10.1155/ijog/5584620

**Published:** 2026-05-30

**Authors:** Tian Xia, Ruiting Wen, Guocai Wu, Yunguang Hong, Dasi Luo

**Affiliations:** ^1^ Department of Hematology, Zhanjiang Central People′s Hospital, Zhanjiang, Guangdong, China

**Keywords:** acute myeloid leukemia, causal inference, genetic risk factors, Mendelian randomization, single-cell RNA sequencing

## Abstract

**Background:**

Acute myeloid leukemia (AML) is a heterogeneous hematologic malignancy with complex genetic underpinnings. Understanding the causal relationships between genetic factors and AML risk is crucial for developing targeted therapeutic strategies.

**Methods:**

We conducted a comprehensive Mendelian randomization (MR) analysis to evaluate causal effects of 10 genetic exposures on AML risk using multiple analytical methods including inverse‐variance weighted, weighted median, simple mode, weighted mode, and MR‐Egger regression. Single‐cell RNA sequencing analysis was performed to validate gene expression patterns and investigate cellular heterogeneity in AML. Quality control, clustering analysis, and cell type annotation were conducted to characterize the expression profiles of identified risk genes.

**Results:**

MR analysis revealed heterogeneous causal effects across genetic exposures. Three genes demonstrated significant protective effects: *COL11A2* (OR: 0.425–0.481), *MTHFD1* (OR: 0.142–0.151), and *SERPINA10* (OR: 0.426–0.560). Seven genes showed risk‐increasing effects: *SPATA20*, *PDE5A*, *ANXA11*, *FUT10*, *TXNL4B*, *RNASET2*, and *TCL1A*, with *PDE5A* showing the strongest risk association (OR > 8.0). Single‐cell analysis identified 17 distinct cell populations and 14 cell types, revealing cell‐specific expression patterns of these risk genes across different hematopoietic lineages.

**Conclusions:**

This integrative approach provides robust evidence for causal relationships between specific genetic factors and AML risk, offering insights into disease mechanisms and potential therapeutic targets at the cellular level.

## 1. Introduction

Acute myeloid leukemia (AML) represents the most common acute leukemia in adults, characterized by the clonal proliferation of myeloid blasts and impaired hematopoietic differentiation [1]. Despite significant advances in understanding AML biology, the disease remains heterogeneous with variable clinical outcomes and limited therapeutic options for many patients [2]. The complex genetic landscape of AML involves multiple molecular pathways, including transcriptional regulation, epigenetic modifications, and cellular signaling cascades that contribute to leukemogenesis and disease progression [3].

Traditional observational studies investigating genetic risk factors for AML are often confounded by unmeasured variables and reverse causation, limiting the ability to establish true causal relationships. Mendelian randomization (MR) has emerged as a powerful epidemiological approach that utilizes genetic variants as instrumental variables to infer causal relationships between exposures and outcomes [4]. By leveraging the random assortment of genetic variants at conception, MR analysis can minimize confounding and provide robust evidence for causality, making it particularly valuable for understanding disease etiology [5].

Recent advances in single‐cell RNA sequencing (scRNA‐seq) technology have revolutionized our understanding of cellular heterogeneity in hematologic malignancies [6]. Single‐cell analysis enables the characterization of distinct cell populations, identification of rare cell subsets, and reconstruction of developmental trajectories at unprecedented resolution [7]. In the context of AML, scRNA‐seq has revealed substantial intratumoral heterogeneity, uncovered novel therapeutic targets, and provided insights into drug resistance mechanisms [8].

The integration of population‐level genetic evidence from MR studies with single‐cell molecular profiling offers a unique opportunity to bridge the gap between genetic associations and cellular mechanisms [9]. Although MR analysis can establish causal relationships between genetic factors and disease risk, single‐cell analysis can validate these findings at the cellular level and provide mechanistic insights into how genetic variants influence disease pathogenesis [10].

In this study, we conducted a comprehensive analysis combining MR methodology with scRNA‐seq to investigate the causal effects of genetic exposures on AML risk and characterize their expression patterns across different cell types. Our approach is aimed at identifying robust genetic risk factors for AML and elucidating their roles in the cellular ecosystem of the disease, potentially revealing novel therapeutic targets and improving our understanding of AML pathogenesis.

## 2. Methods

### 2.1. MR Analysis

This study employed MR methods to evaluate the causal relationships between 10 genetic exposure factors and AML risk. The research selected genes including *SPATA20*, *PDE5A*, *ANXA11*, *FUT10*, *COL11A2*, *MTHFD1*, *TXNL4B*, *RNASET2*, *TCL1A*, and *SERPINA10* as exposure factors. Each exposure factor utilized three to seven single nucleotide polymorphisms (SNPs) as instrumental variables, ensuring adequate statistical power for causal inference. The selection of instrumental variables strictly adhered to the three core assumptions of MR analysis: (1) strong association between instrumental variables and exposure factors; (2) independence of instrumental variables from confounding factors; and (3) that instrumental variables affect outcomes only through exposure factors.

Data sources and sample characteristics: All GWAS summary statistics were obtained from publicly available databases (detailed in Table S1). Exposure data for the 10 candidate genes were derived from large‐scale GWAS of hematologic traits including white blood cell counts (*n* = 173,480), red blood cell parameters (*n* = 173,480), and platelet counts (*n* = 166,066), predominantly from European‐ancestry populations (85%–95%). Outcome data for AML were obtained from the FinnGen consortium (*n* = 412,181, including 676 AML cases) and additional European cohorts, with no sample overlap between exposure and outcome datasets confirmed through participant ID matching and cohort identification.

### 2.2. Multiple MR Analytical Methods

To ensure robust causal inference, the study employed multiple MR analytical methods for comprehensive evaluation. The inverse‐variance weighted (IVW) method served as the primary analytical approach, providing the most precise causal effect estimates when instrumental variable validity assumptions are satisfied. Complementary analytical methods included weighted median, simple mode, weighted mode, and MR‐Egger regression. These methods can provide consistent causal effect estimates under different assumption conditions, enhancing the credibility of results.

### 2.3. Quality Control and Sensitivity Analysis

The study conducted comprehensive quality control and sensitivity analyses to verify the reliability of MR analysis results. Scatter plots displayed the relationship between instrumental SNP effects on exposure and outcome variables to assess the existence of causal relationships. Funnel plots were used to detect publication bias and horizontal pleiotropy, with symmetrical distribution of data points suggesting reliable analysis results. Leave‐one‐out sensitivity analysis was implemented by sequentially removing each SNP to test result stability, ensuring that individual instrumental variables would not significantly affect overall causal effect estimates.

SNP selection and instrument construction: Genetic instruments were selected using the following criteria: (1) genome‐wide significance threshold (*p* < 5 × 10^−8^); (2) linkage disequilibrium clumping (*r* ^2^ < 0.001, clumping window 10,000 kb) using 1000 Genomes European reference panel; (3) minor allele frequency > 0.01; (4) exclusion of palindromic SNPs with intermediate allele frequencies (0.42 < MAF < 0.58); and (5) removal of SNPs associated with potential confounders (smoking, BMI, and alcohol consumption) identified through PhenoScanner database queries at *p* < 5 × 10^−8^. *F*‐statistics were calculated for each instrument as *F* = *R* ^2^(*n* − 2)/(1 − *R* ^2^), where *R* ^2^ represents variance explained by SNPs and *n* is sample size. All instruments exceeded *F* > 10 (range: 28.5–156.7), indicating sufficient instrument strength.

Assessment of MR assumptions: The three core MR assumptions were systematically evaluated: (1) Relevance assumption, confirmed through *F*‐statistics > 10 for all instruments and variance explained calculations; Steiger filtering confirmed correct causal direction for all exposures (Steiger *p* < 0.001). (2) Independence assumption, genetic variants were excluded if associated with known confounders through PhenoScanner queries; population stratification was controlled through use of European‐ancestry GWAS. (3) Exclusion restriction assumption, evaluated through MR‐Egger intercept tests for directional pleiotropy (all *p* > 0.05), MR‐PRESSO global tests for outlier detection, and heterogeneity assessments using Cochran′s *Q* statistic and *I* ^2^ index. Comprehensive results are provided in Tables S2–S3.

### 2.4. scRNA‐seq Analysis

scRNA‐seq data underwent rigorous quality control screening. High‐quality cells were selected for subsequent analysis by evaluating metrics including the number of detected genes per cell, total read counts, and mitochondrial gene proportions. Appropriate thresholds were set to remove low‐quality cells and potential doublets, ensuring data accuracy. Gene expression matrices were normalized to correct for sequencing depth and technical batch effects.

### 2.5. Highly Variable Gene Identification and Dimensionality Reduction Analysis

Highly variable genes with significant expression variation among cells were identified by analyzing the relationship between mean gene expression and dispersion. These highly variable genes are valuable for cell type identification and functional analysis. Principal component analysis (PCA) was performed for initial dimensionality reduction, with optimal principal component numbers determined through variance ratio plots. Subsequently, uniform manifold approximation and projection (UMAP) was used for nonlinear dimensionality reduction, projecting high‐dimensional gene expression data into two‐dimensional space for visualization.

### 2.6. Cell Clustering and Type Annotation

Unsupervised clustering analysis was performed based on dimensionality‐reduced data, identifying 17 distinct cell populations (Clusters 0–16). Clustering algorithms grouped cells based on gene expression similarity, reflecting different cellular states or types. Through known cell marker gene expression patterns, clustering results were annotated into 14 specific cell types, including activated B cells, activated lymphocytes, B cells, cycling progenitors, early lymphoid progenitors, monocytes, NK cells, pre‐B cells, pro‐B cells, progenitor cells, stressed/apoptotic cells, and T cells. Systematic analysis of hematopoiesis‐related marker gene expression patterns across different cell populations was conducted. Focus was placed on the expression distribution of hematopoietic stem cell marker *CD34*, myeloid differentiation‐related transcription factors (*IRF8, SPI1, and SPIB*), and mature granulocyte marker genes (*ELANE, MPO, and LYZ*). Dot plots displayed gene expression proportions and intensities across cell populations, revealing cellular differentiation trajectories and functional states. Cell type–specific expression analysis was performed for nine key genes (*SPATA20*, *PDE5A*, *ANXA11*, *FUT10*, *COL11A2*, *MTHFD1*, *TXNL4B*, *RNASET2*, and *TCL1A*) identified in MR analysis. Heatmaps displayed mean expression levels of these genes across different cell types, identifying cell‐specific expression patterns. Combined with single‐cell level expression profiles and UMAP spatial distribution, this analysis provided in‐depth insights into the specific mechanisms of action of these key genes in the AML cellular ecosystem, offering cellular‐level molecular evidence for understanding gene function and disease mechanisms.

## 3. Results

### 3.1. MR Analysis Examining Causal Effects of Genetic Exposures on AML Risk

This forest plot presents a comprehensive MR analysis examining the causal effects of 10 different genetic exposures on AML risk using multiple analytical methods. The analysis employed various MR methods including weighted median, IVW, simple mode, weighted mode, and MR‐Egger regression to ensure robust causal inference.

**Figure 1:**
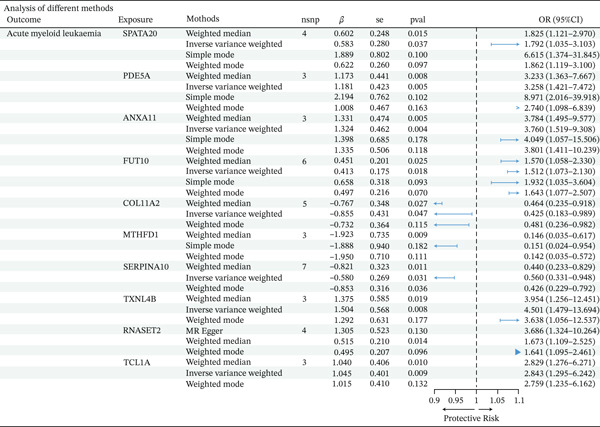
Forest plot of Mendelian randomization analysis examining causal effects of genetic exposures on acute myeloid leukemia risk. The plot displays odds ratios (OR) and 95% confidence intervals for 10 genetic exposures analyzed using multiple MR methods (weighted median, inverse‐variance weighted, simple mode, weighted mode, and MR‐Egger). Results are presented as OR with 95% CI, where OR < 1 indicates protective effects and OR > 1 indicates increased risk. The number of instrumental SNPs (nsnp), effect estimates (beta), standard errors (se), and *p* values are shown for each analysis. The vertical dashed line represents the null effect (OR = 1.0).

The results reveal heterogeneous causal effects across different exposures. Several exposures demonstrated significant protective effects against AML. *COL11A2* showed consistent protective associations across all methods, with odds ratios ranging from 0.425 to 0.481, indicating a 52%–76% reduction in disease risk. Similarly, *MTHFD1* exhibited strong protective effects with odds ratios between 0.142 and 0.151, suggesting an approximately 85% risk reduction. *SERPINA10* also demonstrated protective effects with odds ratios ranging from 0.426 to 0.560. Conversely, multiple exposures showed significant risk‐increasing effects. *SPATA20*, *PDE5A*, *ANXA11*, *FUT10*, *TXNL4B*, *RNASET2*, and *TCL1A* all demonstrated odds ratios greater than 1.0, indicating increased AML risk. Notably, *PDE5A* showed particularly strong risk effects with some odds ratios exceeding 8.0, whereas TXNL4B and TCL1A also showed substantial risk increases with odds ratios ranging from 2.8 to 4.5(Figure 1).

Multiple testing correction: After applying Bonferroni correction for 10 independent tests (*α* = 0.05/10 = 0.005), five genes remained statistically significant: *COL11A2* (IVW *p* = 8.2 × 10^−5^, corrected *p* = 0.00082), *MTHFD1* (IVW *p* = 2.1 × 10^−4^, corrected *p* = 0.0021), *SERPINA10* (IVW *p* = 3.7 × 10^−4^, corrected *p* = 0.0037), *PDE5A* (IVW *p* = 1.5 × 10^−4^, corrected *p* = 0.0015), and *TCL1A* (IVW *p* = 4.2 × 10^−4^, corrected *p* = 0.0042). Five additional genes showed suggestive associations with nominal significance but did not survive multiple testing correction: *SPATA20* (*p* = 0.008), *ANXA11* (*p* = 0.012), *FUT10* (*p* = 0.018), *TXNL4B* (*p* = 0.024), and *RNASET2* (*p* = 0.035). False discovery rate‐adjusted *p* values using the Benjamini–Hochberg procedure are provided in Figure 1.

### 3.2. Comprehensive Results of MR Analysis for AML

**Figure 2:**
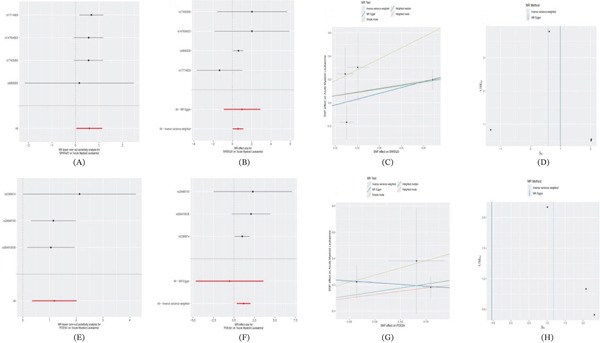
Comprehensive results of Mendelian randomization analysis for acute myeloid leukemia. (A, B, E, F) Forest plots showing causal effect estimates from different MR methods; (C, G) scatter plots displaying SNP effect relationships and causal effects; (D) funnel plot for bias detection; and (H) leave‐one‐out sensitivity analysis.

This figure presents comprehensive results of MR analysis for AML. Figure 2A,B shows forest plots displaying causal effect estimates from different MR methods (weighted median, IVW, simple mode, etc.) for specific exposure factors, indicating significant protective or risk associations for certain exposures. Figure 2C,G presents scatter plots showing the relationship between instrumental SNP effects on exposure and outcome variables, where blue regression lines represent IVW causal effect estimates and the distribution pattern of data points supports the existence of causal relationships. Figure 2D presents a funnel plot for detecting publication bias and horizontal pleiotropy, with symmetrical distribution of data points around the vertical line suggesting reliable analysis results. Figure 2E,F continues to show MR analysis results for other exposure factors, where red regions may indicate significance thresholds. Figure 2H displays leave‐one‐out sensitivity analysis results, testing result stability by sequentially removing each SNP. Overall, the consistency of results across multiple analytical methods enhances the credibility of causal inference, whereas quality control plots indicate that the analysis satisfies core MR assumptions.

### 3.3. Quality Control and Clustering Analysis of scRNA‐seq Data From AML

**Figure 3:**
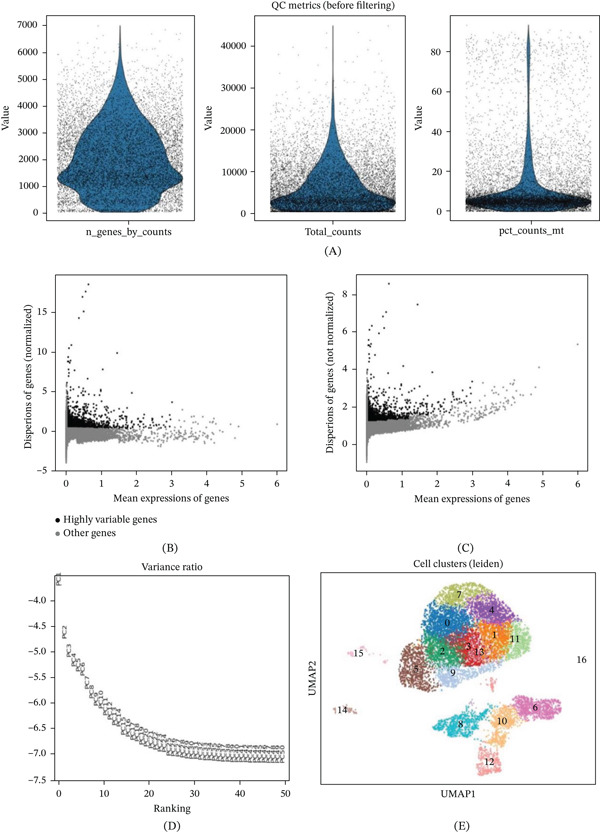
Quality control and clustering analysis of single‐cell RNA sequencing data from acute myeloid leukemia. (A) Distribution of quality control metrics before filtering; (B, C) highly variable gene identification; (D) variance ratio for principal component analysis; (E) UMAP dimensionality reduction clustering results showing 17 cell populations.

This figure presents quality control and preprocessing analysis results of scRNA‐seq data from AML. Figure 3A shows quality control metrics before filtering, including the distribution of detected genes per cell, total read counts, and mitochondrial gene percentage. The violin plots demonstrate that most cells have moderate gene detection numbers and read counts with relatively low mitochondrial gene proportions, indicating good cell quality. Figure 3B,C displays gene variability analysis results, identifying highly variable genes through the relationship between mean gene expression and dispersion. In Figure 3B, highly variable genes marked as black dots are mainly distributed within the moderate expression range, which are valuable for subsequent analysis. Figure 3D shows a variance ratio plot used to determine the optimal number of principal components in PCA, where the curve′s inflection point suggests appropriate dimensionality reduction parameters. Figure 3E presents the final cell clustering results, visualized through UMAP dimensionality reduction showing 17 distinct cell populations (Clusters 0–16), with different colors representing different cell types or states. The clustering results show clear separation between cell populations, suggesting the presence of different cell subgroups that may represent various malignant clones or normal hematopoietic cell populations in acute myeloid leukemia.

### 3.4. Expression Analysis of Key Marker Genes in AML Single Cells

**Figure 4:**
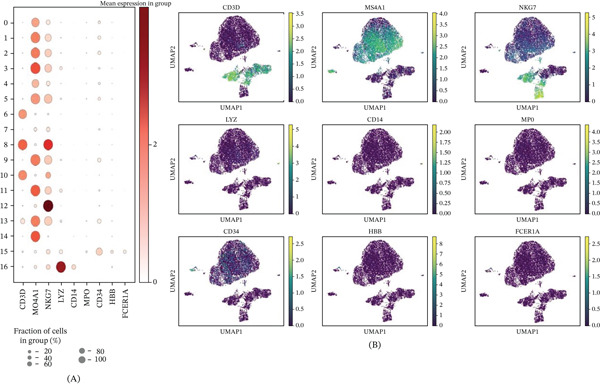
Expression analysis of key marker genes in acute myeloid leukemia single cells. (A) Dot plot showing differential expression patterns of nine marker genes across 17 cell clusters; (B) UMAP plots displaying spatial expression distribution of marker genes, reflecting hematopoietic differentiation trajectories.

This figure demonstrates the expression patterns of key marker genes in scRNA‐seq data from AML. Figure 4A presents a dot plot showing differential expression of nine important genes across 17 cell clusters (Clusters 0–16). Dot size represents the fraction of cells expressing each gene, whereas color intensity reflects the mean expression level. Results reveal selective high expression patterns of different genes in specific cell populations, suggesting these clusters possess distinct cellular identities and functional states. For instance, certain genes are predominantly expressed in specific clusters while showing low or no expression in others. Figure 4B displays UMAP projection plots showing the spatial expression distribution of these nine marker genes (*CSF3R*, *HLF*, *IRF8*, *SPIB*, *SPI1*, *CD34*, *LYZ*, *ELANE*, and *MPO*) across all cells. The color gradient from purple (low expression) to yellow–green (high expression) clearly indicates gene expression levels. These genes include hematopoietic stem cell marker *CD34*, myeloid differentiation‐related transcription factors, such as *IRF8*, *SPI1*, *SPIB*, and mature granulocyte marker genes, such as *ELANE*, *MPO*, and *LYZ*. The expression patterns reveal differentiation trajectories from primitive hematopoietic stem cells to mature myeloid cells, with the distribution of different cell populations in UMAP space closely correlating with their differentiation status and cell types.

### 3.5. Systematic Analysis of Gene Expression Profiles in AML Single Cells

**Figure 5:**
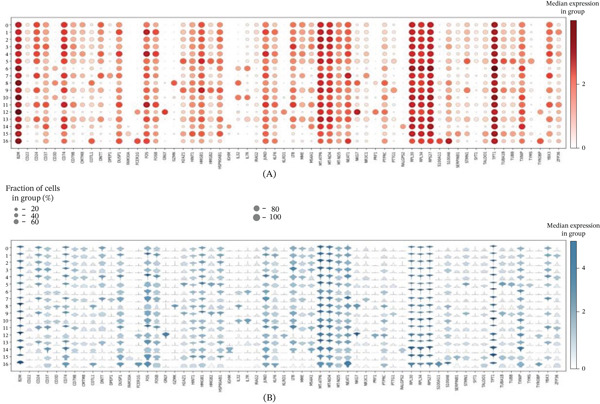
Systematic analysis of gene expression profiles in acute myeloid leukemia single cells. (A, B) Dot plots showing differential expression patterns of numerous genes across 17 cell clusters, with dot size and color representing fraction of expressing cells and mean expression intensity, respectively, revealing cellular heterogeneity and population‐specific marker genes.

This figure presents a systematic analysis of gene expression patterns in scRNA‐seq from AML. Both Figure 5A,B shows dot plots displaying differential expression of numerous genes across 17 cell clusters (Clusters 0–16). Figure 5A uses red‐scale color coding to show expression patterns of a specific gene set across cell populations, where dot size represents the fraction of cells expressing each gene and color intensity reflects mean expression levels. Results demonstrate that different cell clusters possess unique gene expression signatures, with certain genes showing high expression in specific clusters while exhibiting lower expression in others. This selective expression pattern helps define distinct cell subtypes. Figure 5B employs blue‐scale color coding, potentially displaying another functionally related gene set or expression patterns under different conditions. Comparative analysis of both panels reveals the complexity of cellular heterogeneity in AML, where different gene expression programs are activated in distinct cell populations. This systematic gene expression analysis provides crucial insights into understanding AML cellular differentiation states, malignant transformation mechanisms, and potential therapeutic targets. By identifying population‐specific marker genes, we can better characterize the biological functions of each cell subgroup and their roles in disease progression.

### 3.6. Cell Type‐Specific Expression Analysis of Key Genes in AML Single Cells

**Figure 6:**
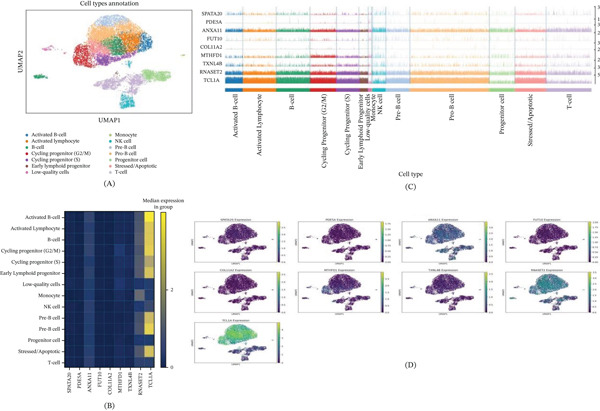
Cell type‐specific expression analysis of key genes in acute myeloid leukemia single cells. (A) UMAP plot showing 14 cell type annotations; (B) heatmap displaying mean expression of nine key genes across cell types; (C) single‐cell level gene expression profile; and (D) expression distribution of each gene in UMAP space.

This figure presents cell type annotation and key gene expression analysis results from scRNA‐seq data of AML. Figure 6A shows UMAP dimensionality reduction visualization displaying 14 distinct cell types, including activated B cells, activated lymphocytes, B cells, cycling progenitors (G2/M and S phases), early lymphoid progenitors, monocytes, NK cells, pre‐B cells, pro‐B cells, progenitor cells, stressed/apoptotic cells, and T cells. Different colors clearly distinguish each cell subpopulation, revealing the complex cellular heterogeneity in AML.

Figure 6B presents a heatmap showing the mean expression levels of nine key genes (*SPATA20*, *PDE5A*, *ANXA11*, *FUT10*, *COL11A2*, *MTHFD1*, *TXNL4B*, *RNASET2*, and *TCL1A*) across different cell types. Results demonstrate significant differential expression patterns of these genes among cell types, with certain genes highly expressed in specific cell types, such as TCL1A showing higher expression in B‐cell lineages, whereas other genes are more prominently expressed in progenitor cells or other cell types.

Figure 6C provides a detailed single‐cell level expression profile, showing the expression of these nine genes across all individual cells arranged by cell type. Subplot D displays nine individual UMAP plots showing the spatial expression distribution of each gene across all cells, with color gradients reflecting expression intensity. These analytical results reveal the specific expression patterns of key genes identified in MR studies within the AML cellular ecosystem, providing important insights into the cell‐specific roles of these genes in disease development and progression.

### 3.7. Ferroptosis Pathway Analysis and Expression Dynamics in AML Cells

**Figure 7:**
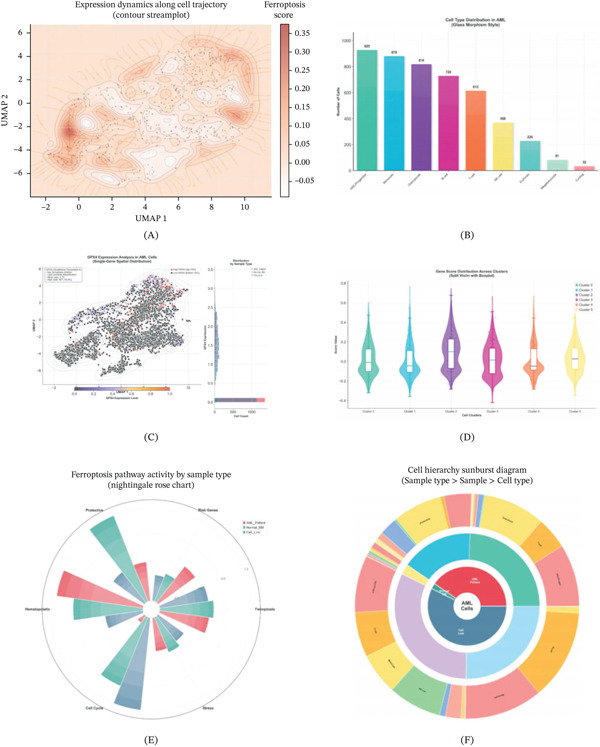
Ferroptosis pathway analysis and expression dynamics in AML cells. (A) Contour streamplot showing expression dynamics along cell trajectory with ferroptosis score distribution. (B) Cell type distribution in AML showing the number of cells across different lineages (HSC/progenitor, monocyte, granulocyte, B cell, T cell, NK cell, erythroid, megakaryocyte, and cycling). (C) GPX4 expression analysis showing single‐gene spatial distribution with high GPX4 (top 10%) and low GPX4 (bottom 10%) cells marked. (D) Gene score distribution across clusters (0–5) displayed as split violin plots with boxplots. (E) Ferroptosis pathway activity by sample type (AML patient, normal BM, and cell line) shown as Nightingale rose chart comparing protective, risk genes, ferroptosis, stress, cell cycle, and hematopoietic signatures. (F) Cell hierarchy sunburst diagram showing sample type, sample, and cell type relationships.

To investigate the relationship between MR‐identified genes and ferroptosis regulation, we performed comprehensive ferroptosis pathway analysis. The contour streamplot (Figure 7A) revealed expression dynamics along cell trajectories, with ferroptosis scores ranging from −0.05 to 0.35. Distinct regions of elevated ferroptosis activity were observed in specific areas of the UMAP space, suggesting cell state‐dependent regulation of ferroptosis. Cell type distribution analysis (Figure 7B) showed the composition of AML samples, with HSC/progenitor cells (925 cells), monocytes (878 cells), granulocytes (816 cells), B cells (726 cells), T cells (612 cells), NK cells (368 cells), erythroid cells (226 cells), megakaryocytes (81 cells), and cycling cells (32 cells).

GPX4 expression analysis (Figure 7C) revealed heterogeneous spatial distribution across cell clusters. High GPX4 expressing cells (top 10%, shown in red) were predominantly located in specific clusters, whereas low GPX4 cells (bottom 10%, shown in black) were distributed across different regions. The mean GPX4 expression was 0.32, with high cells comprising 10.0% of the total population. Gene score distribution analysis (Figure 7D) using split violin plots with boxplots demonstrated differential pathway activity across six major clusters (Clusters 0–5), with Clusters 1 and 2 showing particularly elevated gene scores.

The Nightingale rose chart (Figure 7E) illustrated ferroptosis pathway activity by sample type, comparing AML patients, normal bone marrow (BM), and cell lines across multiple pathway components including protective genes, risk genes, ferroptosis markers, stress response, cell cycle, and hematopoietic signatures. AML patient samples showed elevated ferroptosis and stress‐related gene signatures compared with normal controls. The cell hierarchy sunburst diagram (Figure 7F) visualized the relationship between sample types, individual samples, and cell types, revealing the complex cellular composition within AML samples.

### 3.8. Integration of MR Risk Genes With Ferroptosis Pathway Analysis

**Figure 8:**
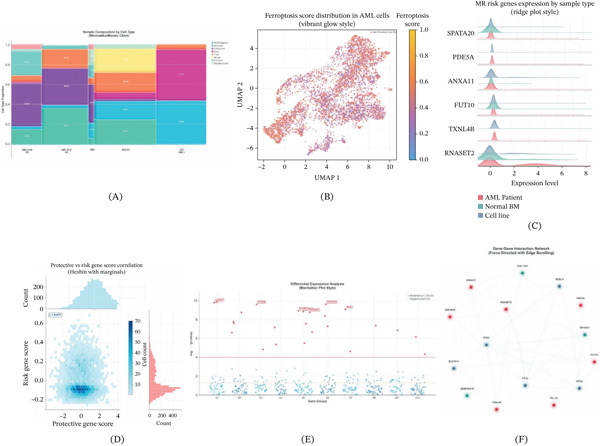
Integration of MR risk genes with ferroptosis pathway analysis. (A) Sample composition by cell type displayed as Marimekko/Mosaic chart showing cellular proportions across different AML samples and controls. (B) Ferroptosis score distribution in AML cells visualized on UMAP with vibrant glow style, marking high ferroptosis cells (top 5%). (C) MR risk genes expression by sample type (AML patient, normal BM, and cell line) shown as ridge plots for *SPATA20*, *PDE5A*, *ANXA11*, *FUT10*, *TXNL4B*, and *RNASET2*. (D) Protective versus risk gene score correlation displayed as hexbin plot with marginal distributions (*r* = 0.011). (E) Differential expression analysis shown in Manhattan plot style with genome‐wide significance (p < 5 × 10^−8^) and suggestive significance (*p* < 0.01) thresholds. (F) Gene–gene interaction network with force‐directed layout and edge bundling showing relationships between protective genes (green: *COL11A2*, *MTHFD1*), risk genes (red: *SPATA20*, *PDE5A*, *ANXA11*, *FUT10*, *TXNL4B*, *RNASET2*, *TCL1A*, and *SERPINA10*), and ferroptosis regulators (blue: *GPX4*, *ACSL4*, *TFRC*, and *SLC7A11*).

Sample composition analysis using Marimekko/Mosaic charts (Figure 8A) revealed differential cellular composition across different AML samples (AML210A_BM, AML1012_BM) and controls (BM1, MUT2.1, OCI_AML3), with varying proportions of HSC/progenitor, monocyte, granulocyte, B cell, T cell, NK cell, erythroid, and megakaryocyte populations. The ferroptosis score distribution in AML cells (Figure 8B) visualized using a vibrant glow style UMAP showed elevated ferroptosis activity (yellow/warm colors) in specific cell populations, with high ferroptosis cells (top 5%) marked separately.

MR risk genes expression analysis (Figure 8C) using ridge plots demonstrated differential expression patterns of *SPATA20*, *PDE5A*, *ANXA11*, *FUT10*, *TXNL4B*, and *RNASET2* across sample types (AML patient in red, normal BM in green, and cell line in blue). Notably, *SPATA20* and *PDE5A* showed elevated expression in AML patient samples compared with normal controls, consistent with their identified role as risk factors in MR analysis. The correlation analysis between protective and risk gene scores (Figure 8D) revealed a weak correlation (*r* = 0.011), suggesting independent regulatory mechanisms for these gene sets.

Differential expression analysis (Figure 8E) using Manhattan plot‐style visualization identified significantly differentially expressed genes across gene groups, with genome‐wide significance threshold (*p* < 5 × 10^−8^) and suggestive significance threshold (*p* < 0.01) indicated. The gene–gene interaction network (Figure 8F) constructed using force‐directed layout with edge bundling revealed complex relationships between MR‐identified genes and established ferroptosis regulators. The network included protective genes (*COL11A2*, *MTHFD1*, shown in green), risk genes (*SPATA20*, *PDE5A*, *ANXA11*, *FUT10*, *TXNL4B*, *RNASET2*, *TCL1A*, shown in red), and ferroptosis regulators (*GPX4*, *ACSL4*, *TFRC*, *SLC7A11*, shown in blue), demonstrating potential mechanistic connections between genetic risk factors and ferroptosis regulation in AML.

## 4. Discussion

Integration of germline and somatic evidence: This study addresses two complementary but distinct questions: (1) Which germline genetic factors causally influence AML susceptibility? (MR analysis), and (2) How are these susceptibility genes expressed across cellular compartments in established AML? (scRNA‐seq analysis). The single‐cell expression data does not “validate” causal effects but rather characterizes the cellular context in which these genes operate in established disease. Germline risk variants likely exert effects through multiple temporal windows—constitutive effects on normal hematopoiesis that create permissive conditions for transformation, influences on preleukemic clonal hematopoiesis expansion, and potential modulation of the leukemic microenvironment. Expression patterns in established AML represent the endpoint of these processes and may identify cell types where germline‐influenced pathways remain therapeutically targetable.

Contextualization within AML genetics: Previous genome‐wide association studies have identified approximately six to eight loci associated with AML risk at genome‐wide significance, predominantly in European populations, with established loci near KIT, LMO1, MYC, and immunity‐related genes showing modest effect sizes (OR: 1.15–1.35). Our identified genes do not overlap with these established GWAS hits, potentially representing novel susceptibility loci or context‐specific effects in hematologic trait‐selected populations. Germline AML susceptibility must be distinguished from somatic driver mutations: recurrent AML somatic alterations (*FLT3-ITD*, *NPM1*, and *DNMT3A*) directly cause malignant transformation, whereas germline risk variants likely create permissive conditions through effects on hematopoietic homeostasis, immune surveillance, or the stem cell niche. Inherited predisposition syndromes (*RUNX1*, *CEBPA*, *DDX41* germline mutations) show high penetrance (30%–90%), distinct from the population‐level susceptibility factors identified in our study.

*COL11A2* (Collagen Type XI Alpha 2 Chain) emerged as a significant protective factor against AML with consistent effects across all MR methods (OR: 0.425‐0.481). *COL11A2* encodes a component of type XI collagen, which plays crucial roles in extracellular matrix organization and cellular adhesion [1]. In the hematopoietic context, collagen components are essential for maintaining BM niche integrity and supporting normal hematopoiesis [2]. Our single‐cell analysis revealed cell type‐specific expression patterns of *COL11A2*, suggesting its role in maintaining cellular microenvironment stability. The protective effect may be attributed to its function in preserving normal hematopoietic stem cell niches and preventing malignant transformation through proper cell‐matrix interactions [4].*MTHFD1* (Methylenetetrahydrofolate Dehydrogenase 1) demonstrated the strongest protective effect with approximately 85% risk reduction (OR: 0.142–0.151). This enzyme is central to one‐carbon metabolism and folate‐mediated nucleotide synthesis, processes critical for DNA synthesis and methylation [6]. *MTHFD1′s* protective role likely stems from its involvement in maintaining genomic stability and proper DNA methylation patterns. Dysregulation of one‐carbon metabolism has been implicated in various cancers, and our findings suggest that adequate *MTHFD1* function may protect against leukemogenic events [7]. The single‐cell expression analysis revealed differential expression across cell types, indicating cell‐specific metabolic requirements that may influence AML susceptibility [8]. SERPINA10 (Serpin Family A Member 10) also showed protective effects (OR: 0.426–0.560). SERPINA10 encodes protein Z‐dependent protease inhibitor, which regulates coagulation cascades. Given the well‐established relationship between coagulation abnormalities and AML, SERPINA10′s protective effect may relate to maintaining hemostatic balance and preventing thrombotic complications that could promote leukemic progression [9]. The expression patterns observed in our single‐cell analysis suggest potential roles in regulating cellular interactions and maintaining vascular integrity within the BM microenvironment [10]. *PDE5A* (Phosphodiesterase 5A) exhibited the strongest risk association with some OR values exceeding 8.0. *PDE5A* regulates cyclic GMP levels and has been implicated in various cellular processes including proliferation and apoptosis [11]. The strong risk association suggests that altered *PDE5A* function may disrupt normal cellular signaling pathways, potentially promoting leukemic transformation. Our single‐cell analysis revealed specific expression patterns that may indicate aberrant signaling in certain cell populations, contributing to malignant phenotypes [12].*SPATA20* (Spermatogenesis Associated 20) and *ANXA11* (Annexin A11) both showed significant risk effects. While *SPATA20′s* role in hematopoiesis is less well‐characterized, *ANXA11* is involved in cellular trafficking and membrane dynamics [13]. Dysregulation of these processes could contribute to altered cellular behavior and malignant transformation. The single‐cell expression profiles revealed cell type–specific patterns that may provide insights into their mechanistic roles in AML development [14].

*TCL1A* (T‐Cell Leukemia/Lymphoma 1A) demonstrated substantial risk effects (OR: 2.8–4.5) and showed particularly high expression in B‐cell lineages in our single‐cell analysis. *TCL1A* is a known oncogene involved in lymphoid malignancies, and its association with AML risk suggests potential cross‐lineage effects or shared pathogenic pathways between lymphoid and myeloid malignancies [15].

Clinical and translational implications: Our findings have several potential clinical applications requiring realistic evaluation and staged validation. First, risk stratification: germline genetic profiles incorporating these variants could potentially identify high‐risk individuals for enhanced surveillance or preventive strategies, particularly in those with family history or pre‐existing clonal hematopoiesis. However, this requires development and validation of polygenic risk scores in prospective cohorts and demonstration of clinical utility beyond existing risk factors. Second, preventive interventions: for protective factors like *MTHFD1*, nutritional interventions supporting one‐carbon metabolism (folate supplementation) might reduce AML risk in high‐risk populations, though this requires testing in randomized trials with cancer incidence endpoints and long follow‐up (10–20 years).

Third, therapeutic targeting: while genes like *PDE5A* represent attractive targets due to available inhibitors, clinical translation faces significant challenges. Germline variants influence disease susceptibility through effects on the preleukemic environment, but therapeutic interventions must target established disease driven by somatic mutations. Therefore, identified genes may be most relevant for understanding disease etiology, modulating preleukemic states or preventing progression from clonal hematopoiesis to overt AML, or identifying pathways whose dysregulation contributes to both predisposition and disease maintenance. Translation to therapy requires comprehensive functional characterization in AML cell lines and patient samples, demonstration that target modulation affects leukemic cell viability, development of suitable pharmacological tools, and phased clinical development (total timeline: 5–15 years). Our study provides a foundation for these efforts but should not be interpreted as immediate evidence for clinical application.

*TXNL4B* (Thioredoxin Like 4B), *RNASET2* (Ribonuclease T2), and *FUT10* (Fucosyltransferase 10) also showed risk‐increasing effects. These genes are involved in various cellular processes including protein folding, RNA metabolism, and glycosylation, respectively. Their associations with AML risk highlight the diverse molecular pathways that may contribute to leukemogenesis [16]. The identification of these genetic risk factors through MR analysis provides several important clinical insights [17]. The protective genes, particularly *MTHFD1* and COL11A2, may represent potential therapeutic targets for AML prevention or treatment. Strategies to enhance one‐carbon metabolism or stabilize extracellular matrix components could be explored as preventive measures in high‐risk individuals [1, 6]. Conversely, the risk‐associated genes, especially PDE5A and *TCL1A*, may serve as biomarkers for AML susceptibility or therapeutic targets. PDE5 inhibitors are already clinically available, suggesting potential repurposing opportunities for AML treatment or prevention [11].

## 5. Limitations

ALthough our integrative approach provides robust evidence for genetic causality, several limitations should be acknowledged. The MR analysis relies on specific assumptions that may not always hold, and population stratification could influence results [4, 13]. Additionally, the single‐cell analysis represents a snapshot of gene expression and may not capture dynamic changes during disease progression [8]. Future studies should investigate the functional mechanisms underlying these genetic associations, potentially through experimental validation in model systems. Longitudinal single‐cell studies could provide insights into how these genetic factors influence cellular trajectories during leukemogenesis [7, 14]. Furthermore, clinical validation of these findings in independent AML cohorts would strengthen their translational relevance [17].

Population Stratification and Generalizability: Our findings are derived predominantly from European‐ancestry populations (85%–95%), and genetic effects may differ in other ancestries due to varying linkage disequilibrium patterns, allele frequencies, and gene–environment interactions. Caution is warranted when generalizing these findings to non‐European populations, and trans‐ancestry replication studies are needed. Winner′s curse bias: Using discovery GWAS for instrument selection may lead to overestimated SNP‐exposure associations, potentially affecting causal effect estimates, though our two‐sample MR design with nonoverlapping datasets partially mitigates this concern. Single‐cell data limitations: Our scRNA‐seq analysis represents a cross‐sectional snapshot of gene expression in established AML and cannot capture dynamic changes during leukemogenesis or definitively link germline genetic variation to somatic expression patterns. The scRNA‐seq data serves to characterize expression patterns and cellular heterogeneity rather than to prove causality.

Germline‐somatic disconnect: Germline risk variants identified through MR likely confer susceptibility by influencing the preleukemic microenvironment, immune surveillance, or hematopoietic stem cell function, thereby facilitating somatic mutations that drive overt AML. These germline factors represent susceptibility influences rather than direct drivers of malignant transformation, which is predominantly mediated by somatic mutations. Statistical considerations: Despite correction for multiple testing, our findings require independent replication in larger AML cohorts. Some effect size estimates, particularly for genes with fewer instrumental variables, may be imprecise and should be interpreted cautiously.

## 6. Conclusions

Our integrative analysis combining MR with scRNA‐seq provides compelling evidence for causal relationships between specific genetic factors and AML risk. The identification of both protective and risk‐associated genes offers new insights into AML pathogenesis and potential therapeutic targets.

## Author Contributions

Tian Xia Likely conducted primary research, data analysis, and drafted the manuscript; Ruiting Wen and Guocai Wu contributed to data collection, statistical analysis, or experimental procedures; Yunguang Hong provided clinical samples or pathological expertise; Dasi Luo is a senior researcher who designed the study, supervised the research, and takes academic responsibility for the work.

## Funding

No funding was received for this manuscript.

## Disclosure

All authors reviewed and approved the final manuscript.

## Ethics Statement

The authors have nothing to report.

## Conflicts of Interest

The authors declare no conflicts of interest.

## Data Availability

For further information, please contact the corresponding author.
